# Salidroside provides neuroprotection by modulating microglial polarization after cerebral ischemia

**DOI:** 10.1186/s12974-018-1081-0

**Published:** 2018-02-09

**Authors:** Xiangrong Liu, Shaohong Wen, Feng Yan, Kuan Liu, Liqiang Liu, Lei Wang, Shangfeng Zhao, Xunming Ji

**Affiliations:** 1 China–America Institute of Neuroscience, Xuanwu Hospital of Capital Medical University, Beijing, 100053 People’s Republic of China; 2 Beijing Key Laboratory of Translational Medicine for Cerebrovascular Diseases, Beijing, 100053 People’s Republic of China; 3 Cerebrovascular Diseases Research Institute, Xuanwu Hospital of Capital Medical University, Beijing, 100053 People’s Republic of China; 4 Department of Neurosurgery, Beijing Tongren Hospital, Capital University of Medical Sciences, Beijing, 100073 People’s Republic of China; 5 Department of Neurosurgery, Xuanwu Hospital, Capital University of Medical Sciences, Beijing, 100053 People’s Republic of China; 6 Cerebrovascular Diseases Research Institute, Xuanwu Hospital of Capital Medical University, 45 Changchun Street, Beijing, 100053 People’s Republic of China

**Keywords:** Salidroside, Inflammation, Microglia, Polarization, Neuron, Stroke, Oligodendrocyte

## Abstract

**Background:**

Following stroke, microglia can be driven to the “classically activated” pro-inflammatory (M1) phenotype and the “alternatively activated” anti-inflammatory (M2) phenotype. Salidroside (SLDS) is known to inhibit inflammation and to possess protective effects in neurological diseases, but to date, the exact mechanisms involved in these processes after stroke have yet to be elucidated. The purpose of this study was to determine the effects of SLDS on neuroprotection and microglial polarization after stroke.

**Methods:**

Male adult C57/BL6 mice were subjected to focal transient cerebral ischemia followed by intravenous SLDS injection. The optimal dose was determined by evaluation of cerebral infarct volume and neurological functions. RT-PCR and immunostaining were performed to assess microglial polarization. A transwell system and a direct-contact coculture system were used to elucidate the effects of SLDS-induced microglial polarization on oligodendrocyte differentiation and neuronal survival.

**Results:**

SLDS significantly reduced cerebral infarction and improved neurological function after cerebral ischemia. SLDS treatment reduced the expression of M1 microglia/macrophage markers and increased the expression of M2 microglia/macrophage markers after stroke and induced primary microglia from M1 phenotype to M2 phenotype. Furthermore, SLDS treatment enhanced microglial phagocytosis and suppressed microglial-derived inflammatory cytokine release. Cocultures of oligodendrocytes and SLDS-treated M1 microglia resulted in increased oligodendrocyte differentiation. Moreover, SLDS protected neurons against oxygen glucose deprivation by promoting microglial M2 polarization.

**Conclusions:**

These data demonstrate that SLDS protects against cerebral ischemia by modulating microglial polarization. An understanding of the mechanisms involved in SLDS-mediated microglial polarization may lead to new therapeutic opportunities after stroke.

## Background

Microglia are the resident macrophages of the brain, with important roles in development, homeostasis, and disease [1, 2]. Under physiologic conditions, microglia are primarily found in the resting state (M0), but are activated into two phenotypes, the “classically activated” M1 and the “alternative activated” M2 phenotypes, following an imbalance to normal physiological conditions [2, 3]. M1 microglia secrete various pro-inflammatory cytokines, such as interleukin (IL)-1β, IL-6, and tumor necrosis factor (TNF)α, which are induced by lipopolysaccharide (LPS) and/or interferon-γ (IFN-γ) [2, 3]. Conversely, M2 microglia produce anti-inflammatory cytokines, such as IL-10 and TGFβ, and are induced by IL-4 and/or IL-13 [2]. After cerebral ischemia, microglia/macrophages are activated: M2 microglia/macrophages promote brain restorative processes, including neurogenesis, axonal regeneration, angiogenesis, oligodendrogenesis, and remyelination; while M1 microglia/macrophages impair neurogenesis and aggravate neurological deficits [2]. Recent evidence suggests that a shift from the M1 phenotype to the M2 phenotype is beneficial for recovery after stroke, and thus may provide novel therapeutic approaches to aide stroke victims [2, 3].

Salidroside (SLDS) is a phenylpropanoid glycoside extracted from the root of *Rhodiola rosea* L and is one of the main active ingredients of this plant. *Rhodiola rosea* grows in high altitudes and cold regions and has been used as a medicine in many European countries and China [4, 5]. Beneficial roles of SLDS have also been reported in aging [5], cancer [6], inflammation [7, 8], oxidative stress [4, 7], and several central nervous system (CNS) diseases, including Alzheimer’s disease [9] and stroke [10, 11]. Recently, SLDS was shown to ameliorate activation of both a microglial [12] and a macrophage cell line [13]. However, to date, the role of SLDS in microglial polarization remains unknown.

The goal of this study was to gain new insight into the medicinal value of SLDS after stroke. The optimal dose of SLDS following middle cerebral artery occlusion (MCAO) in mice was found and the ability of SLDS to regulate microglial polarization was explored both in vivo and in vitro. In addition, the effects of SLDS on primary microglia-mediated inflammation, phagocytosis, oligodendrocyte differentiation, and neuronal death were also investigated. These data provide evidence that SLDS induces neuroprotection by modulating the conversion of M1 microglia to M2 microglia.

## Methods

### Animal model and drug administration

All animal experiments were approved by the Institutional Animal Care and Use Committee of Capital Medical University and in accordance with the principles outlined in the National Institutes of Health Guide for the Care and Use of Laboratory Animals. Transient focal ischemia was induced in male C57/BL6 mice weighing 21–23 g using the intraluminal vascular occlusion method as previously described [14]. Mice underwent MCAO for 1 h and then were reperfused. The mice were randomly assigned to sham-operated, vehicle, and SLDS groups with different doses. Regional cerebral blood flow was measured using laser Doppler flowmetry (PeriFlux System 5000, Perimed, Stockholm, Sweden). Rectal temperature was maintained at 37.0 °C during and after surgery via a temperature-regulated heating pad. SLDS (43866, Sigma, St. Louis, MO, USA) was dissolved in phosphate buffer saline (PBS) for use in animals. Two experimental procedures were initiated:

Experiment 1: To select the optimal dose, SLDS, at 2.5, 5, 10, and 20 mg/kg/day (or PBS) was administered daily via the caudal vein after cerebral ischemia. The first dose of SLDS was given immediately after reperfusion and mice were sacrificed 3 days after MCAO.

Experiment 2: To detect the role of SLDS in microglial polarization after stroke, SLDS was administered once a day for 5 days via the caudal vein. The first dose of SLDS was injected immediately after reperfusion.

### Infarct volume and brain loss analysis

Infarct volume was determined using 2, 3, 5-triphenyltetrazolium chloride (TTC) as previously described [15]. Hematoxylin and eosin (H & E) staining was performed to detect brain loss. The brain loss was measured by subtracting the nonlesioned area of the ipsilateral hemisphere from that of the contralateral hemisphere. The volume of tissue loss was calculated from the lesioned areas in six sections.

### Neurological functional test

To evaluate neurological functional deficits, neurological severity scores were performed at 3 days after MCAO as previously described, by investigators who were blinded to the experimental group assignments [16–18]. The modified neurological severity score is a composite of motor and sensory test. Motor tests were assessed by raising the animal by the tail (normal: 0; flexion of forelimb: 1; flexion of hindlimb: 1; head moved > 10° to vertical axis within 30 s: 1; maximum: 3) and placing the animal on the floor (normal: 0; inability to walk straight: 1; circling toward the paretic side: 2; falling to the paretic side: 3). Sensory tests included tactile response (normal: 0; slowed reaction: 1; no reaction: 2) and proprioceptive response (normal: 0; slowed reaction: 1; no reaction: 2). Tactile response was evaluated by touching the palmar area of forepaw with a sharp needle and proprioceptive response was assessed by pressing a cotton swab against the side of the neck. The overall neuroscore was determined by an investigator blinded to the treatment of the animals.

### Rotarod test

A rotarod test was performed with the Rotamex 5 apparatus (Columbus Instruments, Columbus, OH, USA) as previously described [19]. Briefly, mice were placed on an accelerating rotating rod at an accelerating speed (acceleration from 4 to 40 rpm within 5 min) until the mouse fell onto the platform below, or until the 5 min had elapsed. Each animal underwent three trials daily with an inter-trial interval of 20 min.

### RT-PCR

Total RNA was extracted from microglia or brain tissues using Trizol (Qiagen, Hilden, Germany) according to the manufacturer’s protocol, after which RNA was reverse transcribed into cDNA using Superscript III First-Strand Synthesis SuperMix (Invitrogen, Carlsbad, CA, USA). The resulting cDNA was used for PCR using SYBR GREEN FAST mastermix (Qiagen) in triplicate. The expression of CD16 and CD206 were detected by RT-PCR using the primers: CD16, Forward: 5′-TCAAATCACTTTCTGCCTGCT-3′, Reverse: 5′-CTATTGCTCTCCTCATCCCAT-3′; CD206, Forward: 5′-AGTGATGGTTCTCCTGTTTCC-3′, Reverse: 5′-GGTGTAGGCTCGGGTAGTAGT3′. All other primers for RT-PCR were used as previously described [14, 20–23]. Data collection was performed on the RT-PCR System (Bio-Rad, Hercules, CA, USA). GAPDH was used as an internal control. The relative quantitation value for each gene was performed using the comparative cycle threshold method [24].

### Immunofluorescence staining

Immunofluorescence staining was performed on free-floating sections (25 μm) for tissues or glass coverslips for cell cultures in 24-well plates. Primary microglia, neurons, and oligodendrocytes grown in 24-well plates were fixed with 4% paraformaldehyde. Slides or glass coverslips were washed in PBS and immersed in monkey serum (Jackson Immuno Research Laboratories Inc., West Grove, PA, USA) for 30 min. Primary antibodies included the following: rabbit anti-MAP2 (sc-20172, Santa Cruz Biotechnology, Santa Cruz, CA, USA), rat anti-CD16/32 (553142, BD, Franklin Lakes, NJ, USA), goat anti-CD206 (AF2535, R & D Systems, Minneapolis, USA), rabbit anti-inducible nitric oxide synthase (iNOS, ab15323, Abcam, San Francisco, CA, USA), goat anti-Arg1 (sc-18351, Santa Cruz Biotechnology), rabbit anti-Iba1 (019-19741, Wako, Osaka, Japan), mouse anti-NG2 (MAB5384, Millipore, Billerica, MA, USA), and rabbit anti-MBP (ab40390, Abcam). The nuclei of cells were stained with DAPI (4′6-diamidino-2-phenylindole; Invitrogen) before taking images. Sections or cells were observed under a fluorescence microscope (Carl Zeiss, Jena, Germany) or confocal microscopy (Leica, Wetzlar, Germany).

### Primary culture of microglia, oligodendrocytes, and neurons

Primary rat-enriched microglia were isolated from the whole brains of 1-day-old pups and cultured as previously described [25]. Microglia were shaken off, collected, and reseeded 10 days after initial seeding. Microglia were incubated in DMEM/F12 (Gibco, Life Technologies, Gaithersburg, MD, USA) with 10% fetal bovine serum (Gibco, Life Technologies), and 100 U/ml penicillin/streptomycin (Life Technologies). After microglial collection, oligodendrocytes were shaken off overnight, collected, and incubated in basal chemically defined medium as previously described [26]. NG2 and MBP double immunostaining was performed to identify the stages of oligodendrocyte maturity. All cells were maintained at 37 °C and 5% CO_2_. The M1 phenotype was induced using a combination of LPS (L4391, 100 ng/ml, Sigma) and rat IFN-γ (20 ng/ml, Peprotech, Rocky Hill, NJ, USA) and the M2 phenotype was induced using a combination of rat IL-4 (20 ng/ml, Peprotech) and rat IL-13 (20 ng/ml, Peprotech). Microglia were collected 48 h after initial seeding for mRNA analysis.

Primary cortical neurons were isolated from the brains of E18 rat embryos and incubated in neurobasal medium (Life Technologies) supplemented with 2% B27 (Life Technologies), 2 mM glutamine (Life Technologies) and 100 U/ml penicillin/streptomycin (Life Technologies). The medium was changed every 3 days by replacing two thirds of the medium. Ten days after initial seeding, the purity of neurons was assessed by MAP2 immunostaining (requirement of ≥ 95% purity). At least three independent replicates were performed for all experiments.

### Lactate dehydrogenase (LDH) assay

LDH release was measured using Pierce LDH cytotoxicity kit (Thermo Scientific, Pittsburgh, PA, USA) at 24 h after treatment. Absorbance was read at 450 μm using a Varioskan Flash Reader (Thermo Scientific, Waltham, MA, USA).

### Phagocytosis assay

Microglia were plated in 24-well (3 × 10^5^ cells/well) or 96-well (8 × 10^4^ cells/well) D-lysine (Sigma) coated plates and incubated with different treatments for 48 h. Nile red fluorescent microspheres (Invitrogen) were then added to the cultures for 3 h at a concentration of 0.02% solids. Microsphere counts were performed at 3 h after the addition of the fluorescent microspheres as previously described [14]. Cultures were stained with AlexaFluor488 phalloidin (Invitrogen) and absorbance was read using a Varioskan Flash Reader (Thermo Scientific).

### Assay for pro-inflammatory factors in culture media

Supernatants were collected from microglia with the various treatments stated above at 48 h after treatment. Concentrations of IL-1β, IL-2, IL-6, IL-8, and TNFα were measured with a commercial enzyme-linked immunosorbent assay (ELISA) kit (Neobioscience, Shanghai, China) according to the manufacturer’s instructions. Absorbance was read at 450 μm using a Varioskan Flash Reader (Thermo Scientific).

### Neuron-microglia cocultures

The following two coculture systems were employed: (1) a transwell contact-independent neuron-microglial system and (2) a direct-contact neuron-microglial system; both of which were performed as previously described [14]. To assess neuronal survival in the transwell contact-independent system, neurons were seeded in six-well plates at a density of 8 × 10^5^ per well. Ten days after initial seeding of neurons, activated microglia (4 × 10^5^/well) were added into inserts directly above the neuronal cultures (both cultures shared the same medium). To assess neuronal survival in the direct-contact system, neurons cultured for 10 days in 96-well plates (8 × 10^4^/well) were subjected to either normal conditions or oxygen glucose deprivation (OGD) for 1 h. Primary microglia (4 × 10^4^/well) were then seeded and cultured together with neurons in the presence of different agent combinations in defined culture medium (minimum essential medium containing 10% fetal bovine serum and 10% horse serum). For immunostaining, neurons cultured for 10 days at a density of 2 × 10^5^ per well in 24-well plates were directly cocultured with primary microglia (1 × 10^5^/well).

### Microglia-oligodendrocyte cocultures

Using the transwell coculture system, microglia (1 × 10^5^/well) seeded in inserts were activated by different agent combinations for 48 h, after which the inserts were gently washed twice and added directly above oligodendrocytes (2 × 10^5^/well) cultured in 24-well plates. After 3 days of coculture, microglial inserts were replaced by new 48 h-activated microglial inserts.

### Quantitative MAP2 ELISA

Neurons cultured in six-well plates in the transwell system were collected at 48 h after treatment and lysed. A MAP2 ELISA kit was used (Cusabio, Wuhan, China) to detect the expression of MAP2 as previously described [14].

### Statistics

GraphPad Prism 7.03 software (GraphPad Software Inc., La Jolla, CA, USA) was used for statistical analyses. All data were presented as mean ± standard error of mean (SEM). The Shapiro-Wilk normality test was used to confirm the values derived from a Gaussian distribution. Statistical power was calculated using Gpower 3. Assumptions of equal variance were tested with Brown-Forsythe tests. Significant differences were assessed by Student’s *t* test for two-group comparison and one-way analysis of variance (ANOVA) followed by Tukey’s test or two-way ANOVA followed by Sidak’s test for multiple comparisons. Statistical significance was set at *P* < 0.05.

## Results

### SLDS treatment reduces infarct volume and protects neurological function after MCAO

**Fig. 1:**
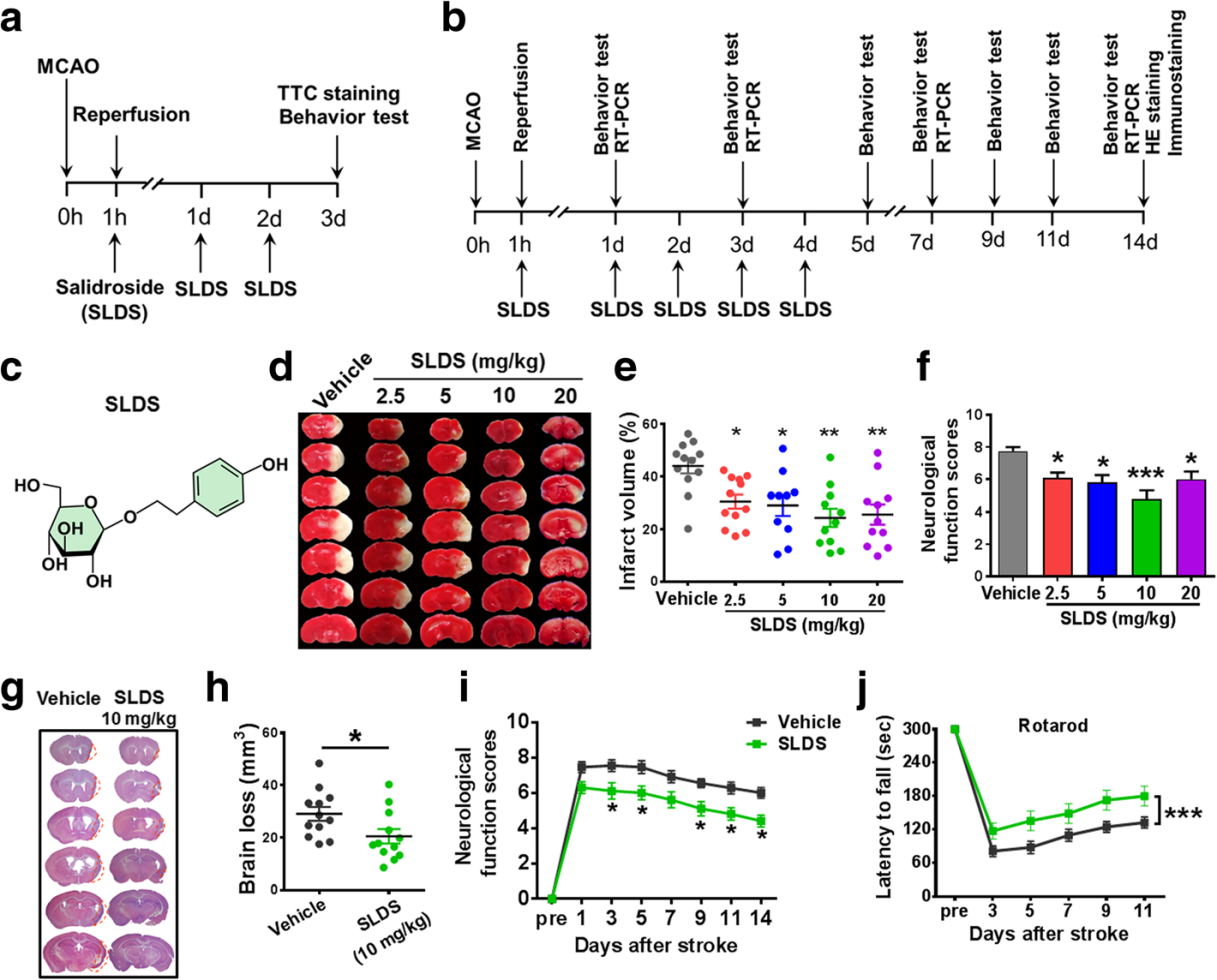
The neuroprotective role of salidroside (SLDS) on MCAO in mice. **a** Timeline for selecting the optimal dose after stroke in mice. SLDS, at 2.5, 5, 10, and 20 mg/kg/day (or PBS), was administered to the mice daily via the caudal vein. **b** Timeline for determining the role of SLDS in microglial polarization after MCAO in mice. SLDS (10 mg/kg/day) or PBS was administered to the mice daily via the caudal vein. **c** Chemical structure of SLDS. **d** SLDS treatment reduced infarct volume. Representative brain slices with infarcts stained by triphenyltetrazolium chloride from each group at 72 h after MCAO. **e** Quantification of infarct volume at 3 days after MCAO. *n* = 10–12 per group. *P < 0.05, **P < 0.01, vs. vehicle group, by one-way ANOVA and Tukey’s test. **f** Quantification of neurological function scores at 72 h after focal ischemia. SLDS treatment improved neurological functions. *n* = 10–12 per group. *P < 0 .05, ***P < 0.001, vs. vehicle group, by one-way ANOVA and Tukey’s test. **g** Representative brain slices were stained to detect brain loss by hematoxylin and eosin (H & E) staining. **h** Quantification of brain loss. SLDS treatment reduced brain loss at 14 days after cerebral ischemia. *n* = 12 per group. *P < 0.05, by Student’s *t* test. **i** Quantification of neurological function scores after MCAO. *n* = 11 in the vehicle group; *n* = 10 in the SLDS group. *P < 0.05 vs. the corresponding days in the vehicle groups, by two-way ANOVA and Sidak’s multiple comparisons test. **j** Quantification of rotarod test after focal cerebral ischemia. *n* = 12 per group. ***P < 0.001, by two-way ANOVA and Sidak’s multiple comparisons test. All data are expressed as mean ± SEM

SLDS was administered as shown in Fig. 1a, b, while the schematic structure of SLDS is shown in Fig. 1c. Infarct volume was significantly attenuated at 72 h after cerebral ischemia with SLDS treatment at incremental doses of 2.5, 5, 10, and 20 mg/kg, respectively (*P* < 0.05, *P* < 0.05, *P* < 0.01, and *P* < 0.01, respectively; Fig. 1d, e). A similar concomitant significant improvement in neurological scores was noted at doses of 2.5, 5, 10, and 20 mg/kg, respectively (*P* < 0.05, *P* < 0.05, *P* < 0.01 and *P* < 0.01, respectively; Fig. 1f). Maximal beneficial effects were observed at a dose of 10 mg/kg and as such, this dose was used in all subsequent experiments to explore the role of SLDS in microglial polarization after MCAO. SLDS treatment decreased the brain loss at 14 days after MCAO (*P* < 0.05; Fig. 1g, h) and improved functional recovery at days 3, 5, 9, 11, and 14 after MCAO compared with controls (all *P* < 0.05; Fig. 1i). To assess general fitness and motor coordination, the rotarod test was performed every 2 days from days 3 to 11 after MCAO. The latency to fall off the rotarod was much longer in the SLDS-treated group compared with the vehicle group (*P* < 0.001; Fig. 1j).

### SLDS promotes M2 macrophage/microglial polarization after MCAO

**Fig. 2:**
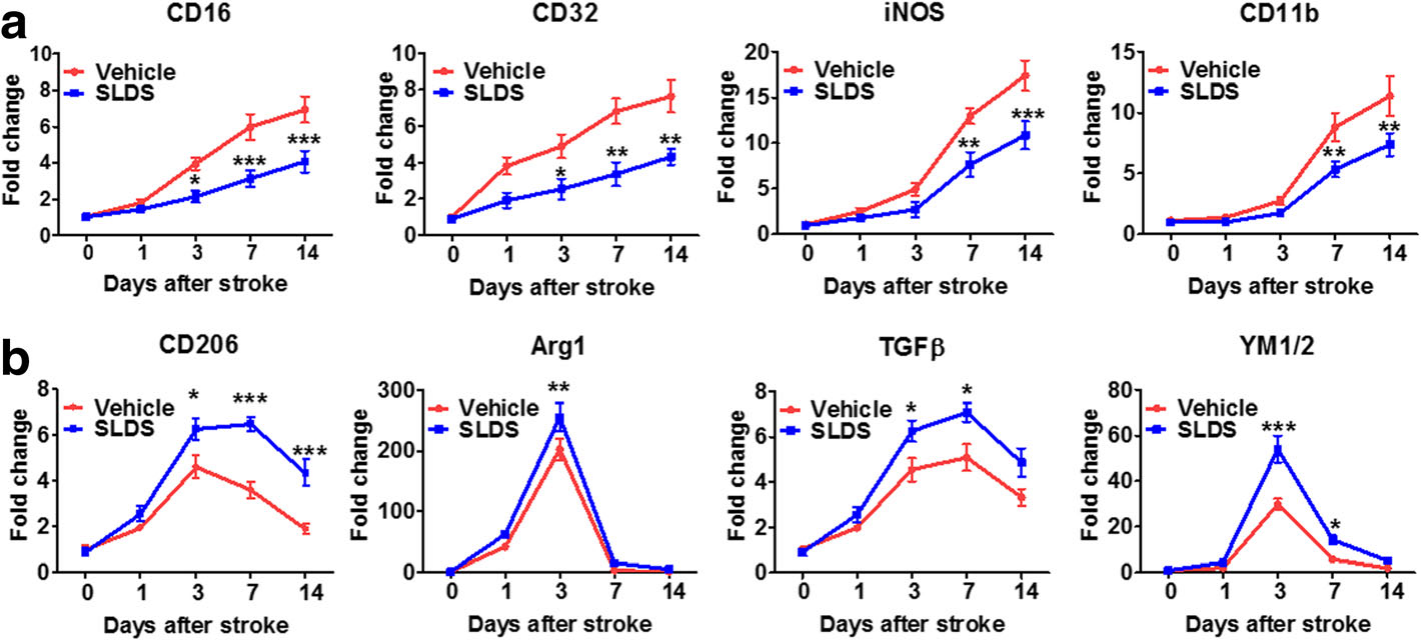
SLDS induces changes in mRNA expression of M1 and M2 polarization markers after MCAO. RT-PCR was performed on mRNA isolated from ischemic brains 1, 3, 7, and 14 days after MCAO or sham operations. **a** SLDS decreased the mRNA expression of M1 polarization markers after MCAO. **b** SLDS increased the mRNA expression of M2 markers after MCAO. *n* = 4 to 5 per group. Data are expressed as mean ± SEM. *P < 0.05, **P < 0.01, ***P < 0.001 vs. the corresponding days in the vehicle groups, by two-way ANOVA and Sidak’s multiple comparisons test

**Fig. 3:**
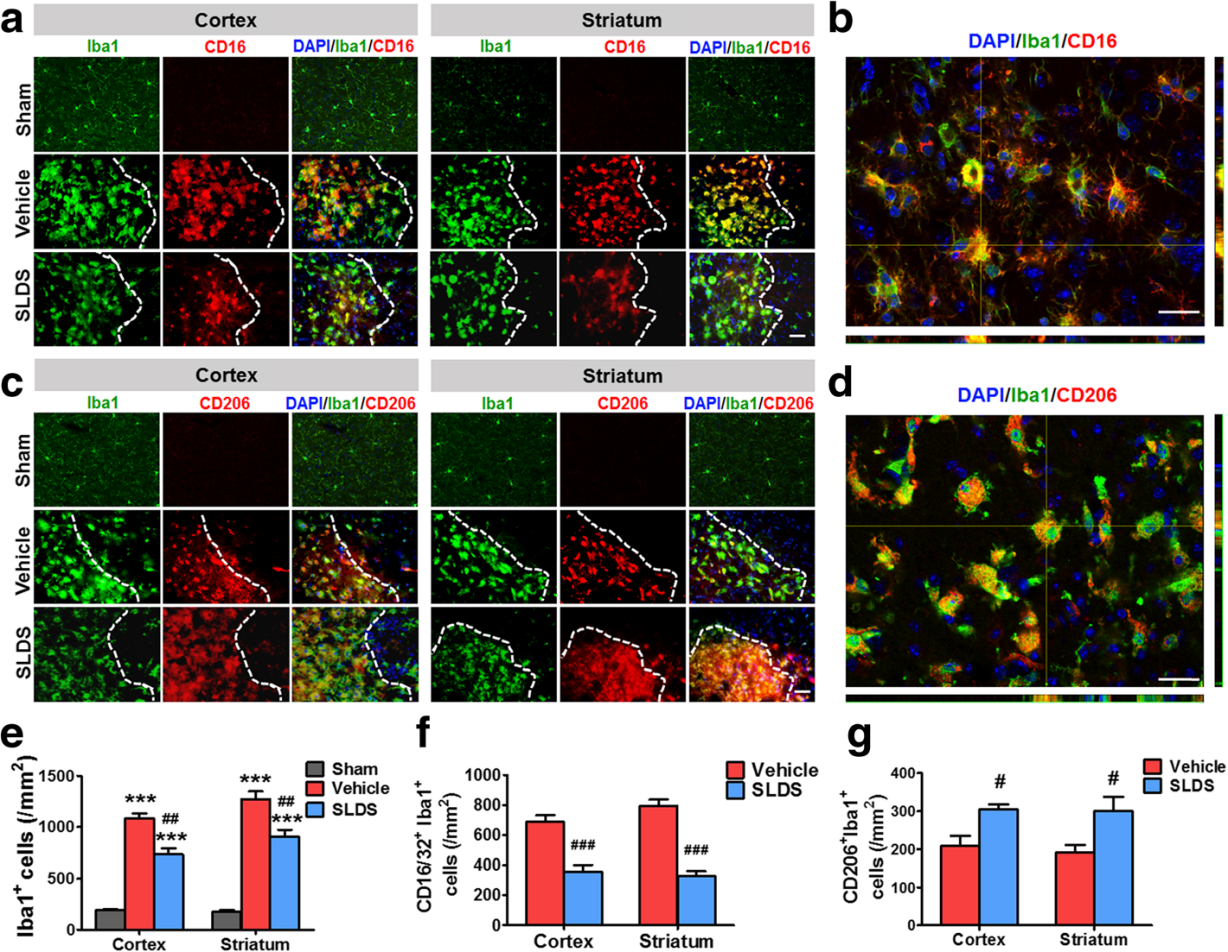
SLDS promotes M2 microglial polarization following MCAO. **a** Representative double immunofluorescence staining for Iba1 (green) and CD16 (red) 14 days after cerebral ischemia in the cortex and striatum. Scale bar, 50 μm. **b** A higher magnification image of CD16/Iba1-positive cells. Scale bar, 25 μm. **c** Representative images of Iba1 (green) and CD206 (red) immunostaining 14 days after cerebral ischemia in the cortex and striatum. Scale bar, 50 μm. **d** A higher magnification image of CD206/Iba1-positive cells. Scale bar, 25 μm. Numbers of Iba1^+^ microglia (**e**), Iba1^+^/CD16^+^ M1 microglia (**f**), and Iba1^+^/CD206^+^ M2 microglia (**g**) were quantified. 4′6-diamidino-2-phenylindole (DAPI, blue). Data are expressed as mean ± SEM; *n* = 5 per group. *P < 0.05, ***P < 0.001 vs vehicle groups, by two-way ANOVA and Tukey’s test

To confirm whether SLDS affects macrophage/microglial polarization, RT-PCR was used to detect mRNA expression levels of M1 phenotype makers (CD16, CD32, iNOS, and CD11b) and M2 phenotype markers (CD206, Arg1, TGFβ, and YM1/2). In comparison with the vehicle groups, mice treated with SLDS showed lower expression of M1 markers (Fig. 2a) and higher expression of M2 markers (Fig. 2b) in the cortex and striatum at 14 days after MCAO. The numbers of the Iba1^+^ microglia were significantly lower in both the cortex (*P* < 0.01) and striatum (*P* < 0.01) of SLDS-treated groups compared with vehicle groups (Fig. 3a–e), which suggested that SLDS alleviated microglial activation following stroke. Consistent with RT-PCR results, SLDS treatment significantly decreased the numbers of Iba1^+^CD16/32^+^ M1 microglia/macrophages (*P* < 0.001 in cortex and striatum, respectively; Fig. 3a–f) and significantly increased the number of Iba1^+^CD206^+^ M2 microglia/macrophages in the cortex (*P* < 0.05) and striatum (*P* < 0.05; Fig. 3c–g) at 14 days after MCAO. These results showed that SLDS treatment promoted M2 macrophage/microglial polarization after stroke.

### SLDS induces a phenotypic change in microglia from the M1 to M2 phenotype in vitro

**Fig. 4:**
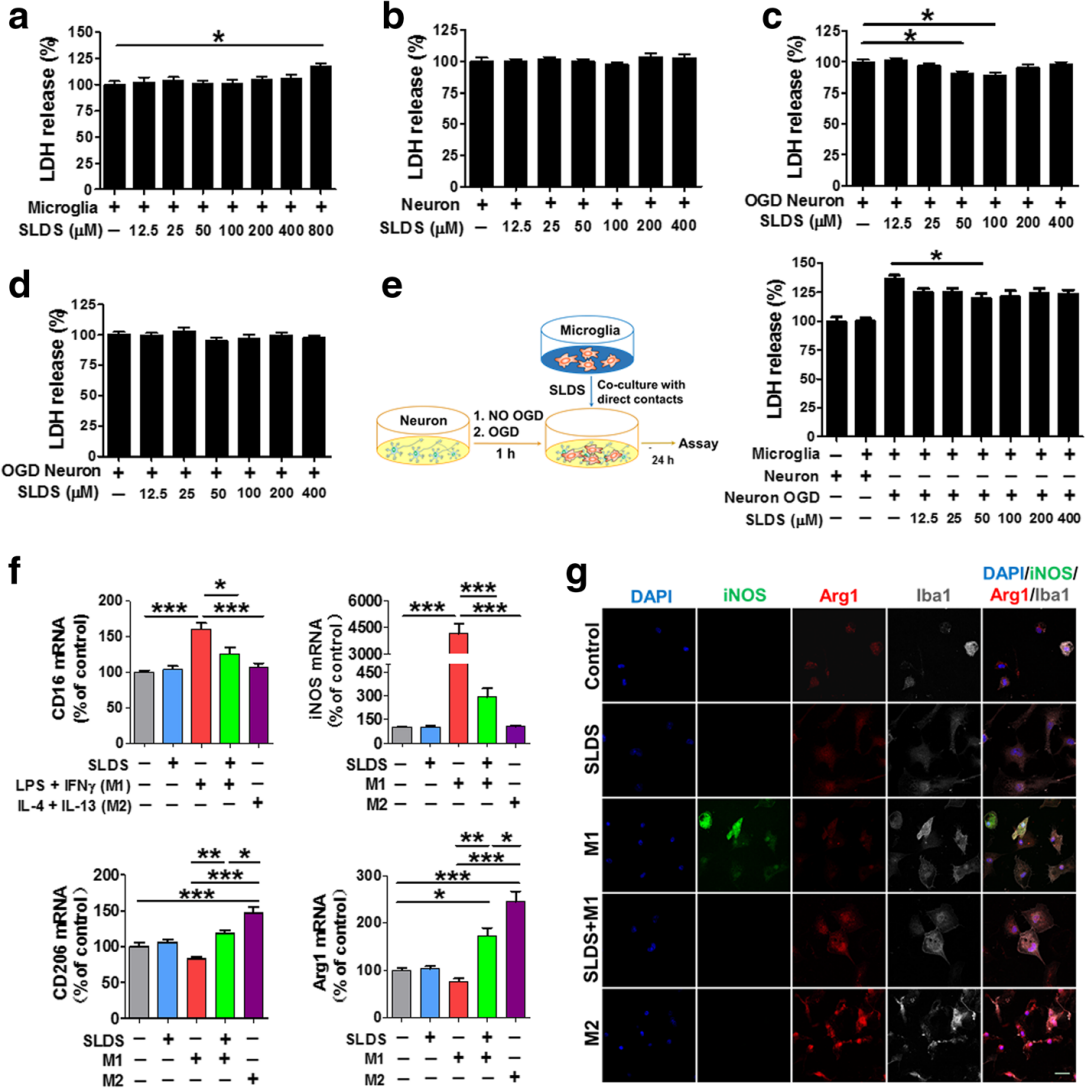
SLDS promotes M2 microglial polarization in vitro. The M1 phenotype was induced using LPS (50 ng/ml) plus IFN-γ (20 ng/ml) and the M2 phenotype was induced using IL-4 (20 ng/ml) plus IL-13 (20 ng/ml). The role of SLDS on cell death of primary microglia (**a**) and primary neurons (**b**); *n* = 12 per group. **c** The role of SLDS in the cell death of primary neurons subjected to 1 h OGD exposure and subsequent 4-h reperfusion. *n* = 15 per group. **d** The role of SLDS in the cell death of primary neurons subjected to 1 h OGD and subsequent 24-h reperfusion. *n* = 12 per group. **e** Neuronal death was quantified by LDH release in primary neuron-microglia cocultures; *n* = 12 per group. **f** mRNA expression of M1 markers (CD16, iNOS) and M2 markers (CD206, Arg1) in primary microglia treated with salidroside or induction factors (or a combination of both). Vehicle was added to non-treated microglia (control); *n* = 3 per group. **g** Representative triple immunofluorescence staining of iNOS (green), Arg1 (red), and Iba1 (gray) in the different treatment groups. Vehicle was added to non-treated microglia (control). DAPI (blue) was used as a nuclear marker. Data are expressed as mean ± SEM. *P < 0.05, **P < 0.01, ***P < 0.001, by one-way ANOVA and Tukey’s test

SLDS, at increasing concentrations, was added into primary microglial and neuronal cultures, and the LDH assay was used to detect subsequent cytotoxicity. SLDS treatment at a concentration of 800 μM significantly elevated LDH release in primary microglia compared with controls (*P* < 0.05; Fig. 4a), while treatments of 400 μM and under did not elicit significant changes in LDH release in either microglia or neurons (Fig. 4a, b). To assess whether SLDS could decrease cerebral ischemia injury in vitro, the LDH assay was performed in primary cortical neurons subjected to an initial 1 h OGD exposure and a subsequent 4- and 24-h reperfusion. As shown in Fig. 4c, d, SLDS (50 and 100 μM) decreased LDH release 4 h after OGD (both *P* < 0.05; Fig 4c), but there were no significant differences between the control group and SLDS groups at increasing concentrations at 24 h after OGD (Fig. 4d).

Furthermore, SLDS treatment (50 μM) of microglial/neuronal cocultures for 24 h resulted in significantly reduced LDH release when neuronal cultures were initially exposed to OGD for 1 h (*P* < 0.05; Fig. 4e). Based on the above results, SLDS treatment at a concentration of 50 μM was used in all subsequent in vitro experiments to explore the role of SLDS in microglial polarization.

Additionally, mRNA levels of CD16 and iNOS were markedly downregulated in M1 microglia treated with SLDS (*P* < 0.05, *P* < 0.001, respectively; Fig. 4f). In contrast, mRNA levels of CD206 and Arg1 were markedly upregulated in microglia after SLDS treatment (both *P* < 0.01; Fig. 4f). Consistent with RT-PCR results, the immunofluorescence assay showed that the expression of iNOS was lower and the expression of Arg-1 was higher in SLDS-treated M1 microglia (Fig. 4g).

### SLDS inhibits the secretion of pro-inflammatory factors and increases phagocytosis of M1 microglia

**Fig. 5:**
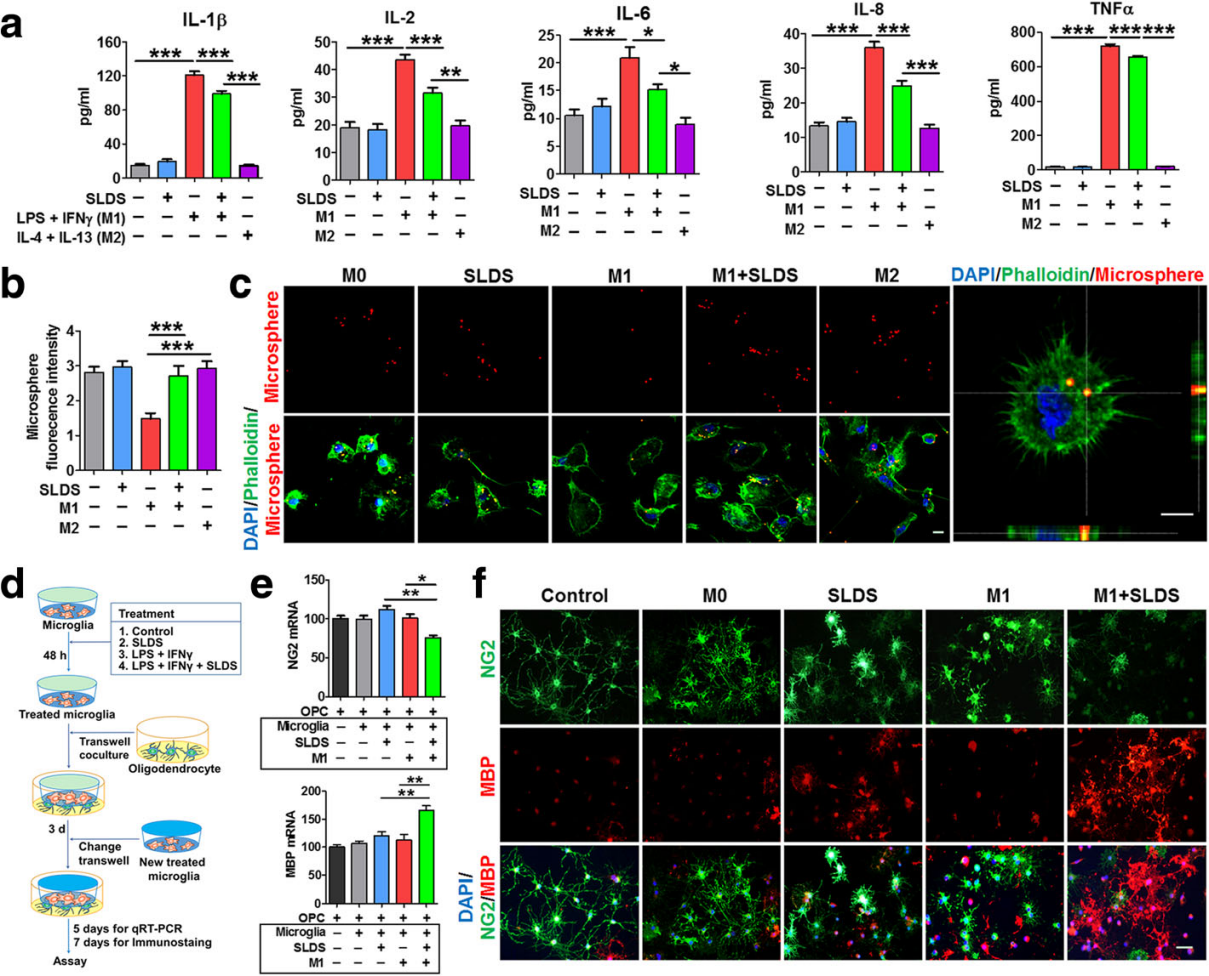
SLDS inhibits inflammatory cytokine secretion, increases phagocytosis in primary microglia, and promotes primary oligodendrocyte differentiation via M2 polarization. The M1 phenotype was induced using LPS plus IFN-γ, and the M2 phenotype was induced using IL-4 plus IL-13. Vehicle was added to non-treated microglia. **a** ELISA results indicated that SLDS inhibited the expression of IL-1β, IL-2, IL-6, IL-8, and TNFα in microglial-conditioned media; *n* = 9–12 per group. **b** Quantification of fluorescent microsphere intensity; *n* = 9 per group. **c** Representative images of microglial phagocytosis detected by fluorescent microspheres. Left scale bar, 10 μm; right scale bar, 5 μm. Phalloidin staining was used to visualize F-actin. **d** In vitro experiments using the transwell contact-independent system. Microglia seeded in inserts were incubated with vehicle or different SLDS treatment combinations for 48 h, after which the inserts were placed over oligodendrocyte cultures. After 3 days in culture, inserts were changed and fresh 48-h-treated microglia inserts were added. Oligodendrocytes were collected 5 or 7 days after the initial microglial insert addition. Vehicle was added to oligodendrocytes alone (control group) or oligodendrocyte-microglia cocultures (M0 group). **e** The expression of NG2 and MBP in the oligodendrocytes cocultured with microglia treated with various concentrations of SLDS (and controls). RT-PCR was performed to detect the expression of NG2 and MBP; *n* = 3 per group. **f** Representative NG2 (green) and MBP (red) staining in the different groups. Scale bar, 50 μm. DAPI (blue) was used as a nuclear marker. Data are expressed as mean ± SEM. *P < 0.05, **P < 0.01, ***P < 0.001, by one-way ANOVA and Tukey’s test

To investigate the effect of SLDS on microglial-mediated inflammatory factor secretion, the levels of pro-inflammatory factors were measured by ELISA. The data showed that SLDS reduced the secretion of the pro-inflammatory cytokines, IL-1β, IL-2, IL-6, IL-8, and TNFα, in M1 microglia (*P* < 0.001, *P* < 0.001, *P* < 0.05, *P* < 0.001, *P* < 0.001, respectively; Fig. 5a). Additionally, M1 microglia treated with SLDS showed a significantly stronger phagocytotic function compared with those not treated with SLDS (*P* < 0.001; Fig. 5b, c).

### M1 microglia treated with SLDS promote oligodendrocyte differentiation

To determine whether microglia treated with SLDS drive oligodendrocyte differentiation, primary microglia were pre-treated with different combinations of medications in a transwell contact-independent system, and then cocultured with oligodendrocytes (Fig. 5d). As shown in Fig. 5e, expression of NG2, a marker of oligodendrocyte progenitor cells, was significantly lower in oligodendrocytes cocultured with SLDS-treated M1 microglia compared with oligodendrocytes cocultured with untreated M1 microglia (*P* < 0.05; Fig. 5e). In contrast, expression of MBP, a marker for mature oligodendrocytes, was significantly higher in oligodendrocytes cocultured with SLDS-treated M1 microglia compared with oligodendrocytes cocultured with untreated M1 microglia (*P* < 0.01; Fig. 5e). Immunostaining results (Fig. 5f) were consistent with RT-PCR data and suggest that SLDS promotes oligodendrocyte differentiation.

### M1 microglia treated with SLDS promote neuronal survival

**Fig. 6:**
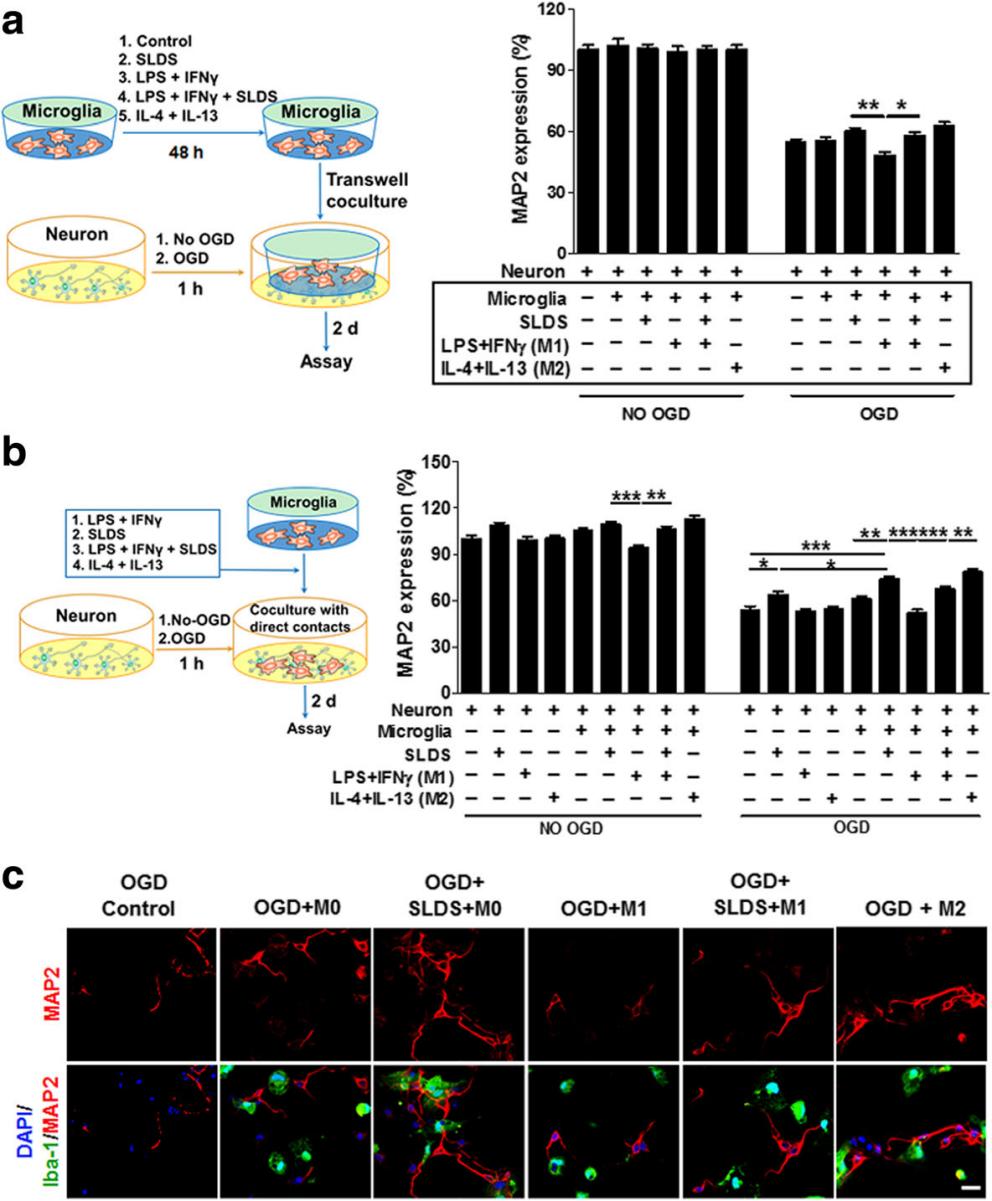
The effect of SLDS-mediated microglial polarization mediated on neuronal survival following OGD. The M1 phenotype was induced using LPS (50 ng/ml) plus IFN-γ (20 ng/ml) and the M2 phenotype was induced by IL-4 (20 ng/ml) plus IL-13 (20 ng/ml). **a** Microglia cultured in transwell plates were treated with different induction factors and/or SLDS for 48 h and conditioned media was removed and added to OGD-treated or untreated neuronal cultures for 48 h (left). Neuronal survival was quantified using MAP2 expression (right); *n* = 12 per group. **b** Microglia were directly added OGD-treated or untreated neurons and cultured for 48 h (left). Neuronal survival was quantified using MAP2 expression (right). *n* = 12 per group. **c** Representative Iba-1 (green) and MAP2 (red) staining of OGD-conditioned neurons cocultured with M0, M1, or M2 microglia in a direct neuron-microglia culture system. Vehicle was added into neuronal cultures and neuron-microglia cocultures without SLDS treatment. Scale bar, 25 μm. DAPI (blue) was used as a nuclear marker. Data are expressed as mean ± SEM. *P < 0.05, **P < 0.01, ***P < 0.001, by one-way ANOVA and Tukey’s test

To gain insight into the effects of SLDS-mediated modification of microglial polarization on neuronal survival, microglia were treated with either SLDS or induction factors (or combination of both), or control, and cocultured with neurons exposed to OGD conditions in a transwell system (Fig. 6a) or in a direct microglial-neuronal contact system (Fig. 6b). As shown in Fig. 6a, in the transwell system, SLDS treatment-enhanced MAP2 expression in neurons exposed to OGD conditions and cocultured with M0 or M1 microglia compared with vehicle-treated M1 microglia (*P* < 0.01, *P* < 0.05, respectively; Fig. 6a). After OGD exposure, SLDS-treated neurons cultured directly with either M0 microglia or cultured alone expressed higher levels of MAP2 compared with neurons not exposed to OGD conditions (*P* < 0.05, *P* < 0.01, respectively; Fig. 6b). Results from the direct microglial-neuronal contact cultures indicated that SLDS significantly enhanced MAP2 expression under both normal and OGD conditions in the presence of M1 microglia (*P* < 0.01, *P* < 0.001, respectively; Fig. 6b), and immunostaining of MAP2 (Fig. 6c) confirmed ELISA results. These results suggest that SLDS modulates microglial polarization to enhance neuronal survival.

## Discussion

SLDS is known to protect against stroke and other neurological diseases [5, 9, 10, 27], the effect of SLDS on microglial polarization status after stroke has not previously been investigated. To our knowledge, this is the first study to describe a beneficial effect of SLDS on microglial polarization after stroke.

Cell quantification revealed that there was a delayed peak at 14 days for CD16^+^/Iba1^+^ M1 cells and at 7 days for CD206^+^/Iba1^+^ M2 cells after stroke in mice [28]. Therefore, brain samples at 14 days after ischemia were chosen to define the role of SLDS in microglial polarization in the present work. Our data suggested that SLDS treatment caused a reduction in M1 macrophage/microglia polarization and an increase in M2 macrophage/microglia polarization in both the cortex and striatum of MCAO mice compared to controls. In addition, results from in vitro experiments were in agreement with the above results. Taken together, the findings of this study indicate a role for SLDS in driving M2 polarization. Consistent with our in vivo findings, a previous study reported that SLDS inhibited activation of BV2, a murine microglia cell line [12]. In addition, another study indicated that M1 microglia exhibited reduced phagocytosis and produced pro-inflammatory cytokines, while M2 microglia increased phagocytosis and secreted anti-inflammatory mediators and neurotrophic factors [14, 29]. As has been documented, inflammation is considered to be a vital determinant of outcome following cerebral ischemia injury, which depends partly upon pro-inflammatory factors [29, 30]. Classically, three pro-inflammatory cytokines, IL-1β, IL-6, and TNFα are associated with the inflammatory response following ischemic stroke [31]. Previous studies indicated that SLDS exhibited anti-inflammatory activities in stroke and other diseases [11, 32, 33]. Here, we confirmed that SLDS inhibits secretion of the pro-inflammatory cytokines in M1 microglia. In addition, M1 microglia treated with SLDS showed increased phagocytosis, similar to levels found in both M0 and M2 microglia, and thus may facilitate brain recovery after stroke.

A previous study showed that SLDS modulated NF-κB and MAPK signaling in LPS-induced BV2 microglial cells [12]. The current results, combined with the fact there are many similarities in microglial differentiation and polarization between humans and rodents [34], point to a potential role for SLDS in the treatment of human patients following stroke, although further clinical research would be needed to confirm SLDS as a treatment option for stroke patients.

Oligodendrocytes and neurons are highly susceptible to ischemic injury, and damage to these cells leads to myelin loss, axonal injury, and neuronal death. Substantial evidence shows that M2 macrophage/microglia drive oligodendrocyte differentiation during central nervous system remyelination, which may promote neurologic recovery [35]. It has been shown that SLDS attenuates arthritis-induced and beta amyloid-induced cognitive deficits [7, 36]. Indeed, our data showed that M1 microglia treated with SLDS promoted oligodendrocyte differentiation via a shift from the M1 to the M2 phenotype, which suggest that SLDS may promote remyelination following neurologic diseases. Previous studies showed that SLDS protected neurons by inhibiting autophagy [37], apoptosis [38] and oxidative stress [39]; which is in agreement with our results whereby SLDS treatment improved the survival of OGD-conditioned neurons cultured with or without microglia.

Our study has several limitations. First, the effects of SLDS on oligodendrocyte differentiation via microglial polarization were limited to normal conditions, rather than in OGD conditions. Moreover, it was not determined whether SLDS-mediated microglial polarization reduced ischemia-induced loss of oligodendrocytes. Second, we did not confirm whether SLDS promoted white matter integrity and long-term functional recovery of white matter after MCAO. Third, the underlying protective mechanism influencing the regulation of microglial polarization induced by SLDS was not explored. The mechanisms of microglial polarization have been investigated extensively [2], but there are no studies defining the role of SLDS on M1/M2 polarization, and its role remains controversial [40]. Further investigation is needed in order to elucidate all of the aforementioned points.

## Conclusions

This study indicates that SLDS modulates microglial polarization and this may contribute to SLDS-induced neuroprotection after MCAO. In addition, SLDS may serve as a promising therapeutic agent to mitigate inflammation and promote functional recovery for stroke and other neurological diseases.

## Abbreviations

ANOVA: Analysis of variance
CNS: Central nervous system
ELISA: Enzyme-linked immunosorbent assay
H & E: Hematoxylin and eosin
IFN-γ: Interferon-γ
IL: Interleukin
iNOS: Inducible nitric oxide synthase
LPS: Lipopolysaccharide
MCAO: Middle cerebral artery occlusion
OGD: Oxygen glucose deprivation
SEM: Standard error of mean
SLDS: Salidroside
TNF: Tumor necrosis factor
TTC: 2, 3, 5-Triphenyltetrazolium chloride
